# The influence of self-esteem and emotional intelligence on addiction to social networks in Peruvian university students

**DOI:** 10.12688/f1000research.163145.1

**Published:** 2025-07-03

**Authors:** Eveling Vargas-Guerrero, Elita Goya-Diaz, Jackeline Morón-Sifuentes, Josue Edison Turpo Chaparro, Isaac Conde, Sanny Huanca-Lopez

**Affiliations:** 1 UPG Educación, Universidad Peruana Union, Lima District, Lima Region, 15462, Peru; 2 Facultad de Ciencias Humanas y Educación, Universidad Peruana Union, Lima District, Lima Region, 15462, Peru; 3 Facultad de Ciencias Empresariales, Universidad Peruana Union, Lima District, Lima Region, 15462, Peru

**Keywords:** Emotional intelligence, self-esteem, Social media, Peruvian university students, social media addiction

## Abstract

**Background:**

The digital age has brought numerous technological advances that have transformed our way of living and relating. Among them, Social media have revolutionized how we communicate and share information. However, there is a silent danger behind this seemingly harmless tool: Social media addiction. For this reason, the objective of this re-search was to determine if emotional intelligence and self-esteem predict addiction to Social media in Peruvian university students.

**Methods:**

Therefore, a cross-sectional predictive design study was developed, in which 289 Peruvian university students participated voluntarily, whose ages fluctuated between 23.61 years with a standard deviation of 5.88 years, of which 54.06% were female and 45.94% were male. In addition, to measure the variables, the Wong-Law Emotional Intelligence Scale (WLEIS), the Rosenberg Self-Esteem Scale – EAR, and the Social media addiction questionnaire (ARS) were used.

**Results:**

Linear regression results show that self-esteem (SELF) is the strongest predictor across all models, with beta coefficients of -0.293 for Social Media Obsession (SMO), -0.225 for Lack of Control, and -0.153 (LAU) for Excessive Social Media Use (USO). Regarding the emotional intelligence dimensions, it is observed that Appraisal of Own Emo-tions (SEA) and Use of Own Emotions (UEO) have weak negative effects on SMO addiction, with beta coefficients ranging from -0.072 to -0.136.

**Conclusions:**

It is concluded that the models explain 17.2% of the variance in SMO, 14.8% in LAU, and 9.2% in USO. These values suggest that, while self-esteem and emotional intelligence influence SMO addiction, other additional factors may also play an important role.

## Introduction

Social media have rapidly impacted contemporary society, significantly transforming how people communicate and becoming essential to daily life ( Miñan Olivos et al., 2023). However, when their use is inappropriate, they can generate uncontrollable changes in the management of the time allocated to them, leading to addiction ( Hawi & Samaha, 2017). If they do not remain connected, those navigating these networks may feel like missing out on something momentous ( Trejos-Gil et al., 2023). Furthermore, currently, social media have a massive global presence, with approximately 4.76 billion users ( Shum, 2023). In Peru, statistics corresponding to 2023 reveal that 93.9% of young people between 19 and 24 use Social media more frequently ( Instituto Nacional de Estadística e Informatica (INEI), 2024). Furthermore, as a result of the Covid-19 pandemic, education was directly affected by both teachers and students ( Jaguaco et al., 2022).

Emotional intelligence is fundamental in regulating digital behaviors ( Valencia-Ortiz et al., 2021). It is defined as the inherent capacity of human beings to identify and express their emotions ( Salovey & Mayer, 1990). Likewise, it is related to the correct management of interpersonal relationships ( Idrogo Zamora & Asenjo-Alarcón, 2021). Correct management of emotions allows the individual to increase positive emotions while avoiding negative ones ( Merino-Soto et al., 2019). Furthermore, its lack of development can lead to isolation, which is a characteristic of addiction to Social media ( Arias Cando & Gavilanes Gómez, 2023).

On the other hand, self-esteem constitutes an individual’s concept of himself, covering two fundamental aspects: self-valuation and recognition of particular abilities ( Orth & Robins, 2014). Furthermore, it is a vital factor in the life of a university student, reflected in their academic achievements ( Vargas & Vásquez, 2019). Consequently, self-esteem is primarily relevant online ( Basauri Delgado, 2023). For this reason, addiction to Social media is characterized by excessive and uncontrolled use of these platforms, which can generate harmful effects on the individual, such as social isolation, distortion between reality and the virtual environment, inattention to other activities, and health problems ( Valencia-Ortiz et al., 2021).

Numerous previous studies have addressed emotional intelligence and social media addiction. Studies confirm an inverse relationship between emotional intelligence and social media addiction. Individuals with low emotional intelligence tend to have higher levels of social media addiction ( Saraiva et al., 2018), and this is exacerbated in people with some type of depressive disorder ( İnaltekin & Yağcı, 2024). Studies consistently show a link between lower emotional intelligence and greater problematic social media use ( Piccerillo & Digennaro, 2024). Emotional intelligence helps improve emotional and social skills and acts as a protector against virtual addictions ( Fernández-Martínez et al., 2023). Studies on 505 female participants reported that excessive online social media use showed an inverse relationship with emotional intelligence ( Tyagi & Meena, 2022). Another study in 466 adults reported that the dimensions of emotional intelligence, emotional management, emotional awareness, and relationship management are affected by problematic social media use ( Kahwagi et al., 2022). A study in 400 participants between the ages of 18 and 25 found that younger people with lower emotional intelligence are more likely to develop addictive behaviors of social media use ( Jarrar et al., 2022).

On the other hand, studies also report an inverse relationship between self-esteem and social media addiction. Research confirms that social media addiction negatively predicts self-esteem levels among university students ( Oleas Rodríguez & López-Barranco Pardo, 2024). Social media addiction is associated with lower self-esteem based on social comparison stigmas, body image issues, or emotional problems ( Colak et al., 2023). Likewise, self-esteem also acts as a mediating factor between social media addiction and other constructs such as depression or anxiety ( Hou et al., 2019). Reports on 311 university students confirmed the effect of social comparison on the relationship between social media and self-esteem, which can be negative or positive ( Kavaklı & Ünal, 2021).

Furthermore, in Peru, negative relationships were identified between addiction to Social media and emotional intelligence in a sample of 279 students ( Alarcon & Salas, 2022). Likewise, Akkus ( Akkus, 2021) determined the existence of a negative and significant correlation between self-esteem and addiction to Social media in a population of 246 students. Bányai et al. ( Bányai et al., 2017) discovered a negative correlation between self-esteem and problematic Social media use in 489 Hungarian participants.

On the other hand, the research by Saraiva et al. ( Saraiva et al., 2018) established a significant negative correlation between emotional intelligence and Social media addiction. Similarly, the study by Rivera and Araujo ( Rivera-Véliz & Araujo-Robles, 2021) found a low, although not significant, correlation between emotional intelligence and Social media addiction variables. Consistent with these findings, Sundvik and Davis ( Sundvik & Davis, 2023) determined a low correlation between Social media addiction and emotional intelligence in their research.

This study is relevant because it aims to show how emotional intelligence and self-esteem predict social media addiction in Peruvian students. In Peru, due to the particularities of the educational system, which has been constantly evolving since 2013 ( Saavedra & Gutierrez, 2020), with a large percentage of rural and indigenous education ( Morales Rocha et al., 2024), and with educational inequalities ( Pasquier-Doumer, 2004), it is important to understand how emotional intelligence and self-esteem predict social media addiction in university students.

### Objectives

The importance of this research lies in addressing the existing knowledge gap about the excessive use of Social media, considering that currently, university students are immersed in these platforms. Consequently, this study provides updated data that will be a factor for new studies based on the variables, contribute to scientific progress, and serve as a reference in future research for psychology professionals and other careers in order to prevent cases of addiction to Social media. For all the above, the objective of the present study was to determine whether emotional intelligence and self-esteem predict addiction to Social media in 2023 Peruvian university students.

## Methods

### Participants

Through non-probabilistic convenience sampling. 289 Peruvian university students from different universities participated in the study. Of the sample, 53.63% were women, and 46.37% were men, ages 23 to 71 years. The inclusion criteria: Students from different faculties and people of legal age. The Exclusion Criteria were Students who responded to less than 100% of the questionnaire and underage university students. In addition to students who did not give informed consent.

Table 1 provides demographic information for the study participants. An average age of 23 to 71 years is reported (M:23.71; SD: 5.85), with a standard deviation of 5.85 years. Regarding sex, 53.63% of the participants were female, while 46.37% were male, reflecting a slight majority of women in the sample. Regarding the university of origin, 63.32% of the participants came from private universities, while 36.68% attended state or public universities.

**Table 1.** Sociodemographic data.

Variables	f	%
Age (M = 23.71; DE = 5.85)		
Female	155	53.63
Male	134	46.37
Public University	106	36.68
Private	183	63.32

### Instruments

The Rosenberg Self-Esteem Scale - EAR ( Atienza et al., 2000) is a self-report scale validated in the Peruvian context ( Ventura-León et al., 2018) which measures self-esteem with the two-dimensional model: positive self-esteem (ω = .803) and negative self-esteem (ω = .723). In addition, it has 10 items with 4 Likert-type response options (“Strongly disagree” = 1 to “Strongly agree” = 4). Likewise, it has scoring categories from 10 to 40. The scale is two-dimensional, as are the original and Spanish versions. Reliability was achieved using Cronbach’s alpha coefficient (α = .86), (α = .87; ω = .88).

The Wong Law Emotional Intelligence Scale - WLEIS ( Wong & Law, 2012), validated in the Peruvian context ( Merino-Soto et al., 2019) comprises 16 items with 7-point Likert-type response options and measures the level of emotional intelligence. Likewise, research shows that its factor structure is divided into four dimensions: 1) Evaluation of one’s own emotions or intrapersonal perception. 2) Evaluation of others’ emotions or interpersonal perception. 3) Use of emotions or assimilation. 4) Regulation of emotions. Con una consistencia interna satisfactoria en sus 4 dimensiones (.87, .90, .84, and .83).

The Social media Addiction Questionnaire (ARS) was developed by Escurra and Salas in 2014 in Peru. It consists of 24 items divided into three dimensions: obsession with Social media, lack of personal control over Social media use, and excessive use of Social media. It uses a five-alternative Likert-type response scale (never = 0, rarely = 1, sometimes = 2, almost always = 3, and always = 4). The authors conducted a confirmatory factor analysis that yielded acceptable goodness-of-fit indices (CFI = 0.95, GFI = 0.92, RMSEA = 0.06), supporting the instrument’s three-factor structure. The questionnaire demonstrated high internal consistency, with Cronbach’s alpha coefficients greater than 0.88 for the total scale and each of the three dimensions ( Escurra Mayaute & Salas Blas, 2014).

### Procedure

Contact was established with several Peruvian university students. They were carefully informed about the details of the study, emphasizing the anonymous, voluntary, and strictly academic nature of their participation. They received a digital informed consent explaining the objectives, procedures to follow, and confidentiality measures implemented. This written consent was requested online from each participant. Without their approval, the survey could not continue. Data was collected using a digital form hosted on the Google Forms platform. This tool allowed remote and controlled access by participants. At the beginning of the form, detailed instructions, informed consent, and a unique code for each participant were presented, thus safeguarding their anonymity. A section of basic sociodemographic data such as age, sex, and type of university was again presented. It was emphasized that all data collected would be used solely for research and that strict anonymity would be maintained. Once the form was completed, participants received a message thanking them for their valuable collaboration with the study.

### Datal analysis

Data analysis was performed using R software version 4.4.1 ( R Core Team, 2024) through the RStudio IDE ( RStudio Team, 2020). At the descriptive level, the mean and standard deviation of the Age variable were calculated. In addition, for the categorical variables Sex and University Management, frequency tables and percentages were obtained using the descr library ( Aquino et al., 2023). For the study variables, an exploratory analysis was performed using box plots to identify and eliminate outliers. Subsequently, descriptive statistics such as mean, standard deviation, skewness, and kurtosis were calculated using the psych library ( Revelle, 2025). At the inferential level, correlation tables were created using apaTables ( Stanley, 2021). Finally, using the seminr library ( Ray et al., 2024), a structural equation model (SEM) was estimated using partial least squares (PLS), specifying both the measurement model and the structural model, and analyzing relationships between the latent variables. Factor loadings, reliability, path effects (structural coefficients), and effect sizes (f ^2^) of the predictors in the model were reported.

## Results

Table 2 shows that all study variables present univariate normality since the coefficients of asymmetry and kurtosis are within the range ± 2.

**Table 2.** Normality analysis of the study variables.

Variable	Min	Max	M	SD	g1	g2
SEA	13	28	21.28	3.37	0.1	-0.42
OEA	10	28	20.96	3.74	-0.04	-0.37
UEO	11	28	20.61	3.54	0	-0.35
ROE	13	28	21.09	3.48	0.11	-0.53
AUTO	17	40	30.49	4.71	0.04	-0.76
OBS	0	35	12.77	8.01	0.54	-0.4
FAL	0	21	9.01	5.14	0	-0.74
USO	0	32	12.68	7.39	0.32	-0.32

Note. Min: Minimum value; Max: Maximum value; M: Mean; SD: Standard deviation; g1: Skewness; g2: Kurtosis; SEA: Assessment of own emotions; OEA: Assessment of others' emotions; UEO: Use of own emotions; ROE: Emotion regulation; SELF: Self-esteem; OBS: Obsession with social media; FAL: Lack of personal control in the use of social media; USE: Excessive use of social media.

Table 3 shows the correlations between the study variables, highlighting the relationships between the dimensions of emotional intelligence, self-esteem, and indicators of social media addiction. A significant positive correlation is observed between self-esteem (AUTO) and the dimensions of emotional intelligence (SEA, OEA, UEO, and ROE), with coefficients between .50 and .53. This indicates that people with higher self-esteem tend to better assess and manage their emotions, both their own and those of others, as well as have a greater capacity for emotional regulation.

**Table 3.** Correlation between the study variables.

	SEA	OEA	UEO	ROE	AUTO
AUTO	.50** [.41; .58]	.53** [.44; .61]	.50** [.41; .58]	.51** [.41; .59]	
OBS	-.29** [-.39; -.18]	-.28** [-.39; -.17]	-.30** [-.40; -.19]	-.27** [-.37; -.16]	-.34** [-.44; -.23]
FAL	-.30** [-.40; -.19]	-.29** [-.39; -.18]	-.30** [-.41; -.20]	-.30** [-.40; -.19]	-.30** [-.40; -.19]
USO	-.25** [-.35; -.14]	-.25** [-.35; -.14]	-.25** [-.36; -.14]	-.24** [-.35; -.13]	-.24** [-.35; -.13]

Note. SEA: Assessment of own emotions; OAS: Assessment of others’ emotions; UEO: Use of own emotions; ROE: Emotion regulation; SELF: Self-esteem; OBS: Obsession with social media; FAL: Lack of personal control in the use of social media; USE: Excessive use of social media.

On the other hand, variables related to social media addiction (OBS, FAL, and USE) present negative correlations with self-esteem and emotional intelligence. Specifically, social media obsession (OBS) shows negative correlations with SELF (-.34) and with all dimensions of emotional intelligence, with values between -.27 and -.30. This suggests that people with a greater obsession with social media tend to have lower self-esteem and a lower capacity for emotional management. Similarly, lack of personal control over social media use (FAL) also has negative correlations with self-esteem (-.30) and with the dimensions of emotional intelligence, with values between -.29 and -.30. This implies that difficulty controlling the time and frequency of social media use is associated with lower emotional appraisal and regulation.

Table 4 shows that all emotional intelligence dimensions (SEA, OAS, UEO, and ROE) exhibit adequate reliability values. Cronbach’s alpha ranges from 0.708 to 0.8, indicating good internal consistency. Furthermore, the composite reliability (CR) values are higher than 0.8, and the average variance extracted (AVE) is above 0.5, suggesting that the items in each scale adequately explain the variance of their construct.

**Table 4.** Reliability of the study variables.

	Alpha	RC	AVE	rhoA
SEA	0.731	0.831	0.552	0.736
OEA	0.8	0.87	0.626	0.801
UEO	0.708	0.818	0.531	0.733
ROE	0.776	0.854	0.595	0.792
AUTO	0.811	0.852	0.387	0.851
OBS	0.911	0.936	0.631	0.955
FAL	0.859	0.894	0.584	0.881
USO	0.938	0.949	0.698	0.947

Note. SEA: Assessment of own emotions; OAS: Assessment of others’ emotions; UEO: Use of one’s own emotions; ROE: Emotion regulation; SELF: Self-esteem; OBS: Obsession with social media; FAL: Lack of personal control in the use of social media; USE: Excessive use of social media.

Regarding self-esteem (SELF), Cronbach’s alpha is 0.811, and the composite reliability reaches 0.852, indicating high reliability. However, the AVE value (0.387) is relatively low, which could suggest that some items do not optimally explain the construct’s variance.

Scales related to social media addiction show even higher levels of reliability. Social media obsession (SMB) presents a Cronbach’s alpha of 0.911, with a composite reliability of 0.936 and an AVE of 0.631, reflecting excellent internal consistency. Similarly, lack of personal control over social media use (LAL) and excessive social media use (USO) have high reliability values, with alphas of 0.859 and 0.938, respectively. The composite reliability for these dimensions exceeds 0.89, and the AVE is greater than 0.5, indicating that the items accurately measure the constructs of social media addiction.

Table 5 presents the results of the linear regressions assessing the impact of self-esteem (SELF) on the dimensions of emotional intelligence. Self-esteem (SELF) is the strongest predictor in all models. This indicates that self-esteem has a positive and significant relationship with all dimensions of emotional intelligence, suggesting that people with higher self-esteem tend to better assess and manage their emotions.

**Table 5.** Regressions of self-esteem and emotional intelligence dimensions as predictors of social media addiction dimensions.

	SEA		OEA		UEO		ROE	
	β	f2	β	f2	β	f2	β	f2
AUTO	0.559	0.486	0.583	0.065	0.577	0.036	0.572	0.016
R ^2^	0.313		0.34		0.333		0.327	

Note. SEA: assessment of own emotions; OAS: assessment of the emotions of others; UEO: use of one's own emotions; ROE: emotion regulation; SELF: Self-esteem.

Regarding the R ^2^ values, it is observed that self-esteem and the emotional intelligence dimensions explain between 31.3% and 34% of the variance in each emotional intelligence dimension. This suggests that a considerable proportion of the variability in these emotional skills can be attributed to self-esteem.

The effect size (f ^2^) for self-esteem varies across models, with the highest effect size for appraisal of others’ emotions (AEO) at 0.065 and the lowest effect size for emotion regulation (ER) at 0.016. This suggests that self-esteem has a particularly strong impact on the perception of others’ emotions, while its influence on emotion regulation is weaker.

Table 6 shows the results of the linear regressions, which analyze the effect of self-esteem (AUTO) and the dimensions of emotional intelligence (SEA, OEA, UEO, and ROE). First, self-esteem (AUTO) is the strongest predictor in all models, with beta coefficients of -0.293 in OBS, -0.225 in FAL, and -0.153 in USO. This indicates that higher self-esteem is associated with lower levels of social media obsession, lack of control, and excessive use. Furthermore, the f ^2^ values for self-esteem are the highest in each model, reinforcing its importance in explaining social media addiction.

**Table 6.** Regressions of self-esteem and emotional intelligence dimensions as predictors of social media addiction dimensions.

	OBS		FAL		USO	
	β	f2	β	f2	β	f2
AUTO	-0.293	0.455	-0.225	0.515	-0.153	0.499
SEA	-0.127	0.003	-0.103	0.001	-0.072	0.001
OEA	0.029	0.000	0.049	0.000	0.006	0.000
UEO	-0.136	0.004	-0.123	0.003	-0.117	0.003
ROE	0.062	0.001	-0.038	0.000	-0.013	0.000
R ^2^	0.172		0.148		0.092	

Note. SEA: assessment of own emotions; OAS: assessment of others' emotions; UEO: use of one's own emotions; ROE: emotion regulation; SELF: Self-esteem; OBS: Obsession with social media; FAL: Lack of personal control in the use of social media; USE: Excessive use of social media.

Regarding the dimensions of emotional intelligence, it is observed that the assessment of one’s own emotions (SEA) and the use of one’s own emotions (UEO) have weak negative effects on social media addiction, with beta coefficients ranging from -0.072 to -0.136. This suggests that better emotional management is related to a lower level of addiction, although its impact is smaller compared to self-esteem.

On the other hand, appraisal of others’ emotions (OEA) and emotion regulation (ER) did not show significant effects on social media addiction, with beta coefficients close to zero and very low effect sizes (f ^2^ ≈ 0.000). This indicates that these dimensions of emotional intelligence do not significantly predict the tendency toward social media addiction.

In terms of explained variance (R ^2^), the models explain 17.2% of the variance in OBS, 14.8% in FAL, and 9.2% in USO. These values suggest that, while self-esteem and emotional intelligence influence social media addiction, other factors may also play an important role.

**Figure 1.**
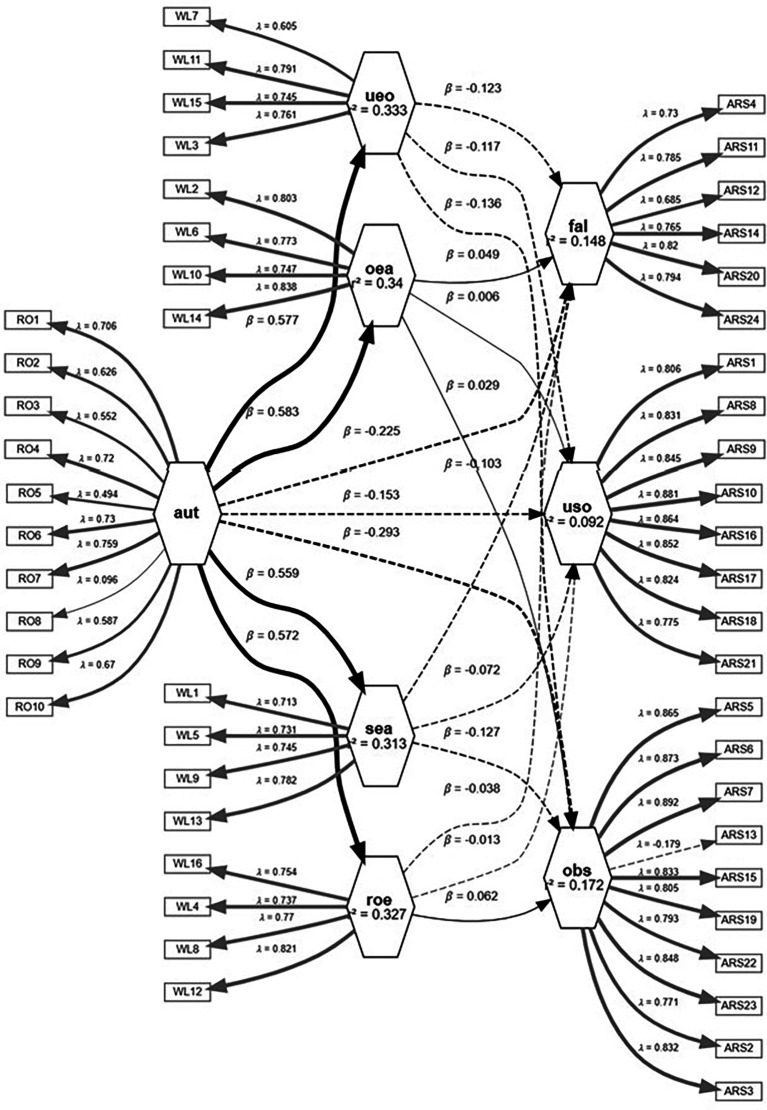
Partial least squares structural equation modeling (PLS-SEM). Note: This figure corresponds to a design by the same authors.

Figure 1 presents a structural equation model (PLS-SEM) that analyzes the relationship between self-esteem, emotional intelligence, and social media addiction. It highlights that self-esteem is the strongest predictor, reducing obsession, lack of control, and excessive social media use. Some dimensions of emotional intelligence also play a role, but with a lesser impact. Furthermore, the model includes factor loadings, which reflect how well the items measure each variable. These loadings are expected to be high (≥0.7), indicating good validity and reliability of the model.

## Discussion

Emotional intelligence and self-esteem are crucial factors for regulating and controlling the use of Social media. They provide people with skills and competencies that allow them to become aware of their behavior in these digital environments ( Garay-Argandona et al., 2021). Veloza ( Veloza Gamba, 2023) maintains that excessive use of Social media negatively affects the development of emotional intelligence and can distort its proper evolution. Martínez et al. ( Martínez-Líbano et al., 2022) report that using Social media negatively affects the health status of university students. For this reason, it is of great relevance to determine whether emotional intelligence and self-esteem are predictors of addiction to Social media.

The findings confirm that self-esteem has a positive relationship with the dimensions of emotional intelligence, meaning that people with higher self-esteem tend to have better assessment, evaluation, and regulation of their emotions. This result is in line with Mayer et al. ( Mayer et al., 2004), where a high degree of emotional attention can negatively affect self-esteem. Good emotional intelligence is associated with better self-esteem ( Calero et al., 2018). Likewise, emotional recovery is related to self-esteem, which ultimately contributes to maintaining a positive image ( Schutte et al., 2002). This relationship has been reported in university students ( Murad, 2021).

On the other hand, self-esteem and emotional intelligence dimensions predict variability in social media addiction. The literature shows that social media addiction decreases self-esteem in university students ( Landa-Blanco et al., 2024). People with low self-esteem are likely to use social media in an extreme way to increase their self-image and self-esteem ( Przepiorka et al., 2024). Higher self-esteem is associated with lower levels of social media addiction ( Mayorca-Martínez et al., 2023). In the case of emotional intelligence, it is inversely related to social media addiction ( Fernández-Martínez et al., 2023). Furthermore, studies show that emotional intelligence can mitigate the relationship between internet addiction and mental health ( Zewude et al., 2024).

The findings confirm that self-esteem has a predictive effect on levels of social media obsession. Theory confirms that low self-esteem can increase people’s vulnerability to developing addictive behaviors online ( Andreassen, 2015). Furthermore, these exposures to virtual content induce negative comparisons that decrease self-esteem and promote negative behaviors in the use of social media ( Vogel et al., 2014). The results confirm the inverse effect of self-esteem on the lack of control in social media use. Studies show that students who have levels of control tend to have higher self-esteem ( Saadat et al., 2012). Likewise, the findings confirm that self-esteem negatively predicts excessive social media use. The literature confirms that higher self-esteem is associated with less addiction ( Rahmawati et al., 2021).

Another finding, albeit with a lesser effect, is that of emotional intelligence in its dimensions of self-assessment of emotions and appropriate use of emotions on social media addiction. College students with low emotional intelligence are prone to problematic social media use ( Kircaburun et al., 2020). Students with poor emotion management skills tend to rely excessively on social platforms ( Hormes et al., 2014).

This study has relevant practical implications, such as the possibility of developing educational programs to control addiction to Social media. Given the constant growth in the use of these platforms, this research constitutes a significant contribution to the existing literature. Social media represent a crucial communication and socialization environment for adolescents. Depending on their use, they can strengthen their ties or contribute to the emergence of specific problems. Given the need to promote responsible and constructive use of these tools,

This study’s limitations could have influenced the results. Furthermore, since this is a self-report, some university students could have responded more honestly due to the time factor. Likewise, the information was acquired virtually, so it could be different when doing it in person. Therefore, conducting more research with representative samples of Peruvian students and other countries is recommended.

We conclude that emotional intelligence and self-esteem are significant constructs to increase or decrease addiction to Social media. According to the results of our research, a moderate negative correlation is observed between Emotional Intelligence and Addiction to Social media. This suggests that people with higher levels of emotional intelligence show less tendency toward Social media addiction. On the other hand, a weak positive correlation is found between Self-esteem and Addiction to Social media. This suggests that people with higher self-esteem may have a slight tendency to show greater addiction to Social media.

## Ethical considerations

This research was approved by the Ethics Committee of the Graduate School, Universidad Peruana Unión on December 11, 2023 (Reference: CE-EPG-000160). It was carried out without specific funding from public, commercial or non-profit organizations. The guidelines for research involving human subjects found in the Declaration of Helsinki will apply. Participants were informed that their participation in this non-experimental research was completely voluntary and that they were free to withdraw at any time without facing sanctions or negative repercussions. Additionally, participants were assured that there were no known risks associated with their participation in the study.

## Informed consent statement

Written Informed consent was obtained from all participants prior to their participation in the study. The full statement for larger samples according to the ethics committee protocol was: The purpose of this questionnaire is to identify factors associated with social media addiction. Your participation is entirely voluntary, and you are not required to complete this survey if you do not wish to. If you decide to participate in this study, please complete the questionnaire. You may also stop completing it at any time if you so choose. I have read the preceding paragraphs and acknowledge that by completing and submitting this questionnaire, I am giving my consent to participate in this study.

## Data Availability

Zenodo: Self-esteem, emotional intelligence and addiction to social networks in Peruvian university students. https://doi.org/10.5281/zenodo.15025316 ( Turpo, J. (2025)) The project contains the following underlying data:
- Data.xlsx. (Anonymised answers to questionnaire) Data are available under the terms of the Creative Commons Attribution 4.0 International license (CC-BY 4.0). 1. Zenodo: Abbreviated consent and study instruments: The influence of self-esteem and emotional intelligence on addiction to social networks in Peruvian university students. https://doi.org/10.5281/zenodo.15052412 ( Turpo Chaparro, J. E. (2025a)). The project contains the following underlying data:
- Informed consent and questionnaires in Spanish Data are available under the terms of the Creative Commons Attribution 4.0 International license (CC-BY 4.0).
2. Zenodo: Abbreviated consent and study instruments: The influence of self-esteem and emotional intelligence on addiction to social networks in Peruvian university students. https://doi.org/10.5281/zenodo.15683281 ( Turpo Chaparro, J. E. (2025b)). The project contains the following underlying data:
- Informed consent and questionnaires translated into English Data are available under the terms of the Creative Commons Attribution 4.0 International license (CC-BY 4.0).
