# Supplementary material for: The predictive rol of self-esteem and emotional intelligence on addiction to social networks in Peruvian university students
Source: F1000Res. 2026 Jul 16;14:647. Originally published 2025 Jul 3. [Version 2] doi: 10.12688/f1000research.163145.2 (PMC13309774; doi:10.12688/f1000research.163145.2)
Supplement: Peer review history

# Reviewer report: Reviewer response for version 1

**DOI:** 10.5256/f1000research.179446.r489669

**Published:** 2026-06-26

**Authors:** Ruby Miranda-Osorio, Maria Teresa hernández

**Affiliations:** 1 Educatión, University Viña del Mar, Viña del Mar, Valparaiso Region, Chile

The study examines the relationship between self-esteem and emotional intelligence with the prediction of social media addiction in 289 Peruvian university students, using a predictive cross-sectional design. Validated instruments were used: Wong Law's Emotional Intelligence Scale, Rosenberg's Self-Esteem Scale and the Social Media Addiction Questionnaire, collecting data in virtual format.

 1.- Clarity and theoretical solidity

 Although the article is coherent, it lacks a more explicit theoretical framework and a problematization with greater depth. It is recommended that the authors integrate a theoretical discussion with greater development that explains the relationship between the variables, especially in the face of findings that seem to contradict previous literature.

 2.- Design and generalization

 The use of non-probabilistic sampling for convenience limits external validity. It is imperative that the authors include a detailed section of Limitations where there is further discussion regarding possible bias affecting extrapolation of results to other university populations.

 3.- Accuracy in the statistical analysis

 Although the use of PLS-SEM is technically adequate, there is evidence of an interpretative inconsistency in the conclusions regarding the relationship between self-esteem and addiction. In addition, the use of the term "predictor" to define relationships in a cross-sectional design should be avoided, since this does not allow causality to be established.

 4.- Transparency and Replicability

 The article stands out for its excellence in the practice of Open Science. The provision of anonymized data, instruments, and consents in Zenodo elevates the ethical and technical integrity of the manuscript.

 Conclusion: To achieve a higher level of rigor, authors must correct inconsistencies and deepen the discussion of methodological limitations derived from their design.

Is the work clearly and accurately presented and does it cite the current literature?

Partly

If applicable, is the statistical analysis and its interpretation appropriate?

Partly

Are all the source data underlying the results available to ensure full reproducibility?

Yes

Is the study design appropriate and is the work technically sound?

Partly

Are the conclusions drawn adequately supported by the results?

Partly

Are sufficient details of methods and analysis provided to allow replication by others?

Yes

Reviewer Expertise:

Tecnología aplicada a la educación; Educación superior; Formación inicial docente; prosecución de estudios

We confirm that we have read this submission and believe that we have an appropriate level of expertise to confirm that it is of an acceptable scientific standard, however we have significant reservations, as outlined above.

# Reviewer report: Reviewer response for version 1

**DOI:** 10.5256/f1000research.179446.r489674

**Published:** 2026-06-23

**Authors:** Boris Córdova-Canova

**Affiliations:** 1 Psicología, Universidad Cesar Vallejo Piura, Piura, Piura, Peru

The manuscript addresses a relevant topic: the relationship between self-esteem, emotional intelligence, and social media addiction among Peruvian university students. The availability of the dataset and supplementary materials is a positive aspect.

 However, several issues should be addressed before the article can be considered fully scientifically sound.

 First, the authors should avoid causal language, such as “influence”, because the study has a cross-sectional predictive design. The findings support associations and statistical prediction, but not causal effects.

 Second, the sample description should be revised. The sex percentages reported in the abstract differ from those reported in the methods section. In addition, the description of age is unclear and should be reported consistently.

 Third, the instruments section requires further development. The authors should report validity and reliability evidence for the Rosenberg Self-Esteem Scale and the Wong-Law Emotional Intelligence Scale from the original studies, as well as from previous validations. In addition, they should estimate and report validity and reliability evidence for these instruments in the present sample.

 Fourth, the statistical approach requires clearer justification. The manuscript refers to linear regressions and PLS-SEM, but the rationale for using PLS-SEM is not sufficiently explained. The authors should describe the model specification, estimation method, assessment criteria for the measurement and structural models, and whether bootstrapping was used. Standard errors, confidence intervals, t-values or p-values for the path coefficients should also be reported.

 Fifth, the reported f² values should be checked, as some appear unusually high in relation to the beta coefficients and R² values. The AVE for the self-esteem construct is also below the conventional .50 threshold, which requires discussion regarding convergent validity.

 Finally, the conclusions should be corrected. The manuscript reports negative correlations and negative beta coefficients between self-esteem and social media addiction indicators. However, the conclusion states that there is a weak positive correlation between self-esteem and social media addiction. This is inconsistent with the reported results.

 Overall, the study addresses an important issue and may contribute to the literature, but revisions are needed in consistency, instrument reporting, methodological justification, statistical reporting, and interpretation of the findings.

Is the work clearly and accurately presented and does it cite the current literature?

Partly

If applicable, is the statistical analysis and its interpretation appropriate?

Partly

Are all the source data underlying the results available to ensure full reproducibility?

Yes

Is the study design appropriate and is the work technically sound?

Partly

Are the conclusions drawn adequately supported by the results?

Partly

Are sufficient details of methods and analysis provided to allow replication by others?

Partly

Reviewer Expertise:

Psychology, psychometrics, quantitative research methods, higher education

I confirm that I have read this submission and believe that I have an appropriate level of expertise to confirm that it is of an acceptable scientific standard, however I have significant reservations, as outlined above.

# Response

**Published:** 2026-07-10

**Authors:** Josue Edison Turpo Chaparro

We thank the reviewers for their valuable suggestions, which substantially improved the manuscript.

**1. First, the authors should avoid causal language, such as “influence”, because the study has a cross-sectional predictive design. The findings support associations and statistical prediction, but not causal effects.**

**Response:** The manuscript was revised to avoid causal language and to ensure consistency with the cross-sectional predictive design.

**2. Second, the sample description should be revised. The sex percentages reported in the abstract differ from those reported in the methods section. In addition, the description of age is unclear and should be reported consistently.**

**Response:** The data were corrected in accordance with the reviewer’s suggestion, and the description of the sample was reported consistently throughout the manuscript.

**3. Third, the instruments section requires further development. The authors should report validity and reliability evidence for the Rosenberg Self-Esteem Scale and the Wong-Law Emotional Intelligence Scale from the original studies, as well as from previous validations. In addition, they should estimate and report validity and reliability evidence for these instruments in the present sample.**

**Response:** We thank the reviewer for this observation. In response, we expanded the Instruments section to include evidence of validity and reliability from the original studies and previous validations of the Rosenberg Self-Esteem Scale and the Wong-Law Emotional Intelligence Scale. We also estimated and reported psychometric evidence for the present sample. Specifically, we reported Cronbach’s alpha, rho_A, composite reliability, and average variance extracted for each construct. The WLEIS dimensions showed adequate reliability and convergent validity in the current sample, whereas the Rosenberg Self-Esteem Scale showed adequate internal consistency but an AVE below the recommended criterion. Therefore, this result was reported transparently and interpreted with caution.

**4. Fourth, the statistical approach requires clearer justification. The manuscript refers to linear regressions and PLS-SEM, but the rationale for using PLS-SEM is not sufficiently explained. The authors should describe the model specification, estimation method, assessment criteria for the measurement and structural models, and whether bootstrapping was used. Standard errors, confidence intervals, t-values or p-values for the path coefficients should also be reported.**

**Response:** We thank the reviewer for this observation. We revised the Data Analysis section to provide a clearer justification for the use of PLS-SEM, specifying the reflective measurement model, the structural paths tested, the estimation procedure, and the criteria used to evaluate the measurement and structural models. We also clarified that the measurement model was evaluated through standardized factor loadings, Cronbach’s alpha, rho_A, composite reliability, and average variance extracted, whereas the structural model was evaluated through standardized path coefficients, R² values, f² effect sizes, and inferential statistics. In addition, a nonparametric bootstrap procedure with 1,000 resamples was conducted, and Tables 5 and 6 were updated to include standard errors, t-values, p-values, and 95% confidence intervals for each structural path.

**5. Fifth, the reported f² values should be checked, as some appear unusually high in relation to the beta coefficients and R² values. The AVE for the self-esteem construct is also below the conventional .50 threshold, which requires discussion regarding convergent validity.**

**Response:** We thank the reviewer for this observation. We reviewed the f² values and found inconsistencies between the standardized path coefficients and the R² values. We recalculated f² using the standard formula, resulting in corrected tables that allow for a more accurate interpretation of predictor effects. Regarding the AVE of self-esteem, we agree that it was below the .50 threshold. The revised manuscript reports this limitation and interprets convergent validity with caution. Although the construct showed good internal consistency, the low AVE indicates that a smaller proportion of variance was captured, which we acknowledged as a psychometric limitation.

**6. Finally, the conclusions should be corrected. The manuscript reports negative correlations and negative beta coefficients between self-esteem and social media addiction indicators. However, the conclusion states that there is a weak positive correlation between self-esteem and social media addiction. This is inconsistent with the reported results.**

**Response:** The conclusion was revised to address the reviewer’s valuable observation and to ensure full consistency with the negative correlations and negative beta coefficients reported in the results.

# Reviewer report: Reviewer response for version 1

**DOI:** 10.5256/f1000research.179446.r408647

**Published:** 2025-09-15

**Authors:** Elizabeth Ifeoma Anierobi

**Affiliations:** 1 Nnamdi Azikiwe University, Awka, Nigeria

I want to commend the efforts of the authors in coming up with this study which has added to the existing knowledge in the field. The background was properly written, though the specific objectively were not captured.  I will suggest that the authors should specify the specific objectives they set out to achieve. They should let their voices be heard in the empirical studies made. In other words, as they present findings, they should make statements that would show their grasp of the studies. 

 The authors should also discuss any theory or theories that their study is anchored on. This theoretical framework is necessary to support the practical implications of the study.

 The discussion of findings is okay but would be necessary for the authors to start off with showing the finding. They should go further to explain whether the finding was expected or not, and thereafter, support their findings with literature already reviewed. If there is a discordant in findings, this should be explained as to why it was so.

Is the work clearly and accurately presented and does it cite the current literature?

Partly

If applicable, is the statistical analysis and its interpretation appropriate?

Yes

Are all the source data underlying the results available to ensure full reproducibility?

No source data required

Is the study design appropriate and is the work technically sound?

Yes

Are the conclusions drawn adequately supported by the results?

Yes

Are sufficient details of methods and analysis provided to allow replication by others?

Yes

Reviewer Expertise:

Educational Psychology, psychosocial and mental wellbeing, adolescents' behaviour and belief system and academic outcomes.

I confirm that I have read this submission and believe that I have an appropriate level of expertise to confirm that it is of an acceptable scientific standard.
